# Developmental Change in Motor Competence: A Latent Growth Curve Analysis

**DOI:** 10.3389/fphys.2019.01273

**Published:** 2019-10-02

**Authors:** Eline Coppens, Farid Bardid, Frederik J. A. Deconinck, Leen Haerens, David Stodden, Eva D’Hondt, Matthieu Lenoir

**Affiliations:** ^1^Department of Movement and Sports Sciences, Ghent University, Ghent, Belgium; ^2^Department of Movement and Sport Sciences, Vrije Universiteit Brussel, Brussels, Belgium; ^3^School of Education, University of Strathclyde, Glasgow, United Kingdom; ^4^Department of Physical Education, University of South Carolina, Columbia, SC, United States

**Keywords:** latent growth curve analysis, motor competence, individual developmental change, children, weight status

## Abstract

**Background:**

The development of childhood motor competence demonstrates a high degree of inter-individual variation. Some children’s competence levels increase whilst others’ competence levels remain unchanged or even decrease over time. However, few studies have examined this developmental change in motor competence across childhood and little is known on influencing factors.

**Aim:**

Using latent growth curve modeling (LGCM), the present longitudinal study aimed to investigate children’s change in motor competence across a 2-year timespan and to examine the potential influence of baseline weight status and physical fitness on their trajectory of change in motor competence.

**Methods:**

558 children (52.5% boys) aged between 6 and 9 years participated in this study. Baseline measurements included weight status, motor competence (i.e., Körperkoördinationstest für Kinder; KTK) and physical fitness (i.e., sit and reach, standing long jump and the 20 m shuttle run test). Motor competence assessment took place three times across a 2-year timespan. LGCM was conducted to examine change in motor competence over time.

**Results:**

The analyses showed a positive linear change in motor competence across 2 years (β = 28.48, *p* < 0.001) with significant variability in children’s individual trajectories (*p* < 0.001). Girls made less progress than boys (β = –2.12, *p* = 0.01). Children who were older at baseline demonstrated less change in motor competence (β = –0.33, *p* < 0.001). Weight status at baseline was negatively associated with change in motor competence over time (β = –1.418, *p* = 0.002). None of the physical fitness components, measured at baseline, were significantly associated with change in motor competence over time.

**Conclusion and Implications:**

This longitudinal study reveals that weight status significantly influences children’s motor competence trajectories whilst physical fitness demonstrated no significant influence on motor competence trajectories. Future studies should further explore children’s differential trajectories over time and potential factors influencing that change.

## Introduction

Motor competence, which reflects the degree of proficient performance in various motor skills as well as the underlying mechanisms (e.g., motor control and coordination; Utesch and Bardid, 2019), is considered a key component in developing a healthy and active lifestyle from early childhood onwards (Stodden et al., 2008; Robinson et al., 2015; Cattuzzo et al., 2016). Various terminologies have been used interchangeably in past literature to refer to this latent concept including “motor proficiency,” “motor performance,” “movement (skill) competence,” “motor ability,” “motor function,” “motor coordination,” and “fundamental movement/motor skills” (Robinson et al., 2015). In alignment with previous studies (e.g., D’Hondt et al., 2013; Robinson et al., 2015; Cattuzzo et al., 2016), this paper uses the term motor competence as a general construct encompassing all forms of goal-directed human movement involving gross body coordination and control.

The role of motor competence in children’s health is well described in the conceptual model of Stodden et al. (2008). This model denotes the relationship between motor competence and physical activity across childhood as well as their interrelations with perceived motor competence, weight status and physical fitness. Physical fitness can be defined as the capacity to perform physical activity and includes components such as cardiorespiratory fitness, musculoskeletal fitness (i.e., muscular endurance and strength) and flexibility (Caspersen and Christenson, 1985; Ortega et al., 2008). As noted in a review article by Robinson et al. (2015), a wealth of predominantly cross-sectional studies show that multiple health-related outcomes, including physical activity (Holfelder and Schott, 2014; Logan et al., 2015) and physical fitness (Hands, 2008; Cattuzzo et al., 2016; Utesch et al., 2019) are indeed positively associated with motor competence. Previous literature has also shown an inverse relationship between weight status and motor competence (D’Hondt et al., 2011; Cattuzzo et al., 2016). However, given the role of motor competence in the development of an active and healthy lifestyle, it is important to understand how motor competence develops across time during childhood. Therefore, we need more longitudinal research that examines the development of motor competence over time and its relationship with other health-related outcomes. For instance, de Souza et al. (2014) compared motor competence, physical activity and physical fitness of children at 6 years of age relative to their physical fitness and physical activity levels at 10 years. The authors found that children who were both fit and active at 10 years of age had a more favorable activity and fitness profile at 6 years and they were also more competent at 6 years compared to their unfit and sedentary peers. Similarly, Henrique et al. (2018) found a significant relationship between motor competence, physical fitness and weight status over time in children aged 6–9 years. Children with consistently better motor competence during the 4 years of follow-up had lower body weight, lower body mass index, lower subcutaneous fat, and higher physical fitness levels at age 6 compared to those with consistently low(er) levels of motor competence (Henrique et al., 2018). However, Henrique et al. (2018) focused on specific changes in motor competence (i.e., stable and unstable trajectories of children scoring below or above a specific percentile) and not on how factors measured at baseline might influence the development of motor competence.

The development of motor competence during childhood is also noted by a high degree of inter-individual variation (Rodrigues et al., 2016). Some children’s competence levels increase whilst others’ competence levels remain unchanged or even decrease over time. However, few studies have taken into account individual change in motor competence development. To our knowledge, Rodrigues et al. (2016) were the first to highlight the importance of individual trajectories in motor competence and physical fitness measures over time. However, the study of Rodrigues et al. (2016) used a test battery that mainly focused on components of physical fitness. Therefore, further research using specific and standardized assessment tools is needed to explore change in actual motor competence over time.

Using latent growth curve modeling, the aim of the present longitudinal study was (1) to gain more insight into children’s individual change in motor competence across a 2-year timespan and (2) to investigate the potential influence of weight status and physical fitness at baseline on changes in motor competence trajectories over time. Based on previous studies (Stodden et al., 2008; Robinson et al., 2015; Rodrigues et al., 2016), it was hypothesized that there would be significant variability in children’s trajectory of motor competence at the individual level. It was also expected that children’s individual trajectory of motor competence would be influenced by age and sex as well as by their weight status and physical fitness level.

## Materials and Methods

### Participants

The present study involved secondary data-analysis from a large-scale longitudinal research project (Vandorpe et al., 2011). These data were collected in primary school children between September 2007 and January 2009. Children were recruited from 13 randomly selected primary schools from all five Flemish provinces and the Brussels-capital region of Belgium. Motor assessments took place annually for three consecutive years (i.e., 2007, 2008, and 2009). Of the original sample of 712 children assessed at each time point, only those children who completed the motor assessments annually and the anthropometric measurements and physical fitness tests at baseline were retained for the purpose of this study. This resulted in a total sample of 558 children (i.e., 293 boys and 265 girls) aged between 6 and 9 years at baseline. Written informed consent was provided for each child by a parent or legal guardian. The study protocol was approved by the Ethics Committee of Ghent University Hospital.

### Procedures

All participants wore light sports clothing and were barefoot during testing, except for the 20 m shuttle run (for which they wore sports shoes). Assessments took place during the physical education classes in the gymnasium of the children’s schools and were conducted three times on an annual basis (during the same season). Test sessions lasted approximately 85 min, with a group of trained examiners conducting the assessments using standardized instructions in accordance with the testing guidelines.

### Measurements

#### Motor Competence

The Körperkoördinationstest für Kinder (KTK) was used to evaluate motor competence. It is a standardized normative product-oriented test battery for 5- to 15-year old children with typical and atypical motor development, which is widely used in Europe (Kiphard and Schilling, 1974, 2007, 2017). The test battery is considered a highly reliable instrument with excellent test–retest reliability for the total raw score (*r* = 0.97), inter–rater reliability and intra–rater reliability for the subtest raw scores (*r* values > 0.85 and *r* values = 0.80–0.96, respectively) (Kiphard and Schilling, 1974, 2007). Content and construct validity have been documented (Kiphard and Schilling, 1974, 2007), and its convergent validity has been established through moderately strong correlations with other standardized assessment tools such as the Bruininks-Oseretsky Test of Motor Proficiency – 2nd Edition (BOT-2; Bruininks and Bruininks, 2005; Fransen et al., 2014), the Motoriktest für Vier- bis Sechsjährige Kinder (MOT 4-6; Zimmer and Volkamer, 1987; Bardid et al., 2016), and the Movement Assessment Battery for Children (M-ABC; Henderson and Sugden, 1992; Smits-Engelsman et al., 1998). The KTK is also considered a very useful motor test battery for longitudinal research because each test item is identical at any age (D’Hondt et al., 2013). The test includes four subtests: (1) balancing backwards (BB) over three beams of decreasing width, (2) moving sideways (MS) with the aid of two wooden boards in 20 s (two attempts), (3) jumping sideways (JS) as often as possible over a bar in 15 s (two attempts), and (4) hopping for height (HH) on one leg over foam squares with consecutive steps of 5 cm per added foam square. For the purpose of the present analysis, the raw scores of each subtest were summed to compute an overall motor competence score. In addition, a standardized motor competence score (or motor quotient, MQ) was also computed using the manual’s normative tables based on the performance of the reference sample (Kiphard and Schilling, 2017). To this end, the raw subtest scores were first transformed into standardized scores adjusted for age (all subtests) and sex (BB, JS, and HH). These standardized subtest scores were then summed and converted into the total KTK MQ.

#### Physical Fitness

Different subtests of the European Test of Physical Fitness (EUROFIT) with adequate reliability were used to assess the health-related components of physical fitness (Council of Europe, 1988). The selection of these tests was based on practical considerations regarding age-appropriateness, user-friendliness and discriminative power among children aged 6–11 years. Cardiorespiratory fitness or endurance was assessed using the multistage fitness test, also known as the EUROFIT 20 m shuttle run test (20 m SR), with an accuracy of 0.5 min. This test involves continuous running between two lines (20 m apart) on time in agreement with recorded beeps. The frequency of the sound signals is gradually increased during this test, requiring children to run faster with each increase in frequency of signals (plus 0.5 km/h each minute from a starting speed of 8.5 km/h). The test was stopped if the subject could no longer keep the pace and failed to reach the line (within 2 m) for two consecutive times and after a warning. The EUROFIT standing long jump test (SLJ) was used as an indicator of musculoskeletal fitness and explosive power (Pillsbury et al., 2013). In this test, participants have to jump as far as possible from standstill and land on both feet. The test is performed twice with the best result used for data analysis with an accuracy of 1.0 cm. Trunk flexibility and hamstrings length were assessed with the EUROFIT sit and reach test (SAR) with an accuracy of 0.1 cm. For this test, participants had to sit on the ground with straight legs, reaching as far as possible with the fingertips to a metal board, with the best score on two consecutive trials used for data analysis.

#### Weight Status

Participants’ body height was measured by using a portable stadiometer with an accuracy of 0.1 cm (Harpenden, Holtain Ltd., Crymych, United Kingdom) and their body weight was determined using a digital scale with an accuracy of 0.1 kg (Tanita, BC-420 SMA, Weda BV, Naarden, Holland). These measures were then used to compute children’s body mass index (BMI, kg/m^2^), which was used as an estimate of weight status.

### Statistical Analysis

Descriptive statistics were calculated for the motor competence scores (i.e., KTK total raw score) at each time point and for the different health-related components of physical fitness (i.e., 20 m SR, SLJ, and SAR) and weight status (i.e., BMI) at baseline using SPSS 25 for Windows.

**FIGURE 1:**
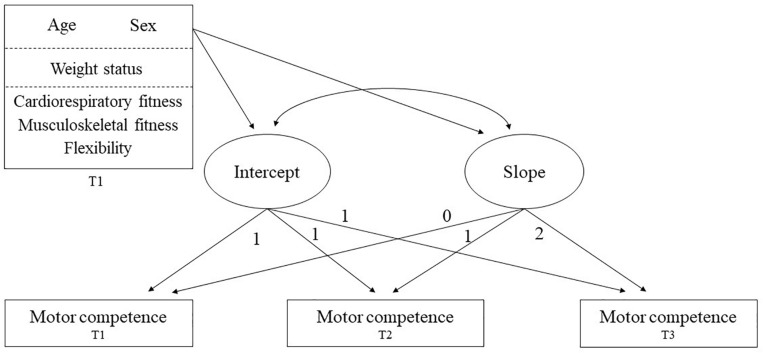
Representation of the latent growth curve model of motor competence measured at three 1-year interval time points (T1, T2, and T3) with age, sex, weight status, and physical fitness as time-invariant covariates measured at baseline (T1). The latent intercept is constant for any child across time points as indicated by the fixed values of 1 for the factor loadings. The latent slope represents a child’s motor competence trajectory with varying values (i.e., 0, 1, and 2) for the factor loadings. The value starts at 0 to allow the mean intercept to be interpreted as the mean motor competence score at baseline (T1). The value increase by 1 indicates an equal amount of time between measurements.

Latent growth curve models (LGCMs; see Figure 1) were conducted to examine change in motor competence over time, summed into an overall motor competence score based on the raw scores on the KTK test items (at each time point). Effects of confounding factors such as sex and age were considered in the analysis. Additionally, effects of participants’ baseline weight status (i.e., BMI) and physical fitness components (i.e., 20 m SR, SLJ, and SAR) on change in motor competence were examined. Maximum likelihood estimation was used for the LGCMs and significance level was set at *p* < 0.05. Different fit indices were used to assess model fit: (1) the chi-square test (χ^2^), (2) the root mean square error of approximation (RMSEA), (3) the standardized root mean square residual (SRMR), and (4) the comparative fit index (CFI). Good model fit is indicated by values < 0.08 (RMSEA), < 0.06 (SRMR) and > 0.90 (CFI; Hu and Bentler, 1999).

A series of LGCMs were run to investigate change in motor competence over time. First, an intercept-only model with the intercept mean and residual variance constrained across time points (Model 1) was run. The intercept variance was then estimated in Model 2. Next, the slope mean and variance were included in Model 3 to estimate change in motor competence over time. Subsequently, sex and age were added to the model (Model 4). Sex was inserted as a dummy variable (i.e., 0 = boy; 1 = girl), whereas age (months) was inserted as a continuous variable and mean centered in the LGCMs. Next, baseline weight status was included as a continuous variable in Model 5 to examine the potential influence on change in motor competence over time. Similarly, 20 m SR, SBJ, and SAR were entered as continuous variables in Model 6 to examine possible effects of baseline physical fitness on motor competence change over time. Both weight status and the three abovementioned physical fitness variables were z-transformed adjusting for age and sex. Finally, a model with only significant effects of baseline weight status and physical fitness components was run (Model 7). All latent growth curve analyses (LGCA) were conducted in R version 3.5.2 using the *lavaan* package (Rosseel, 2012).

**FIGURE 2:**
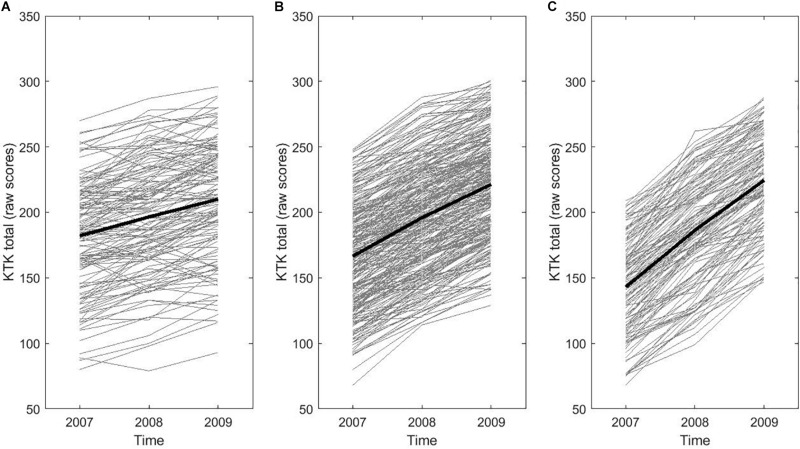
Individual trajectories in motor competence (MC) over time based on the KTK total raw score: representation of **(A)** the lowest (<P25), **(B)** average (P25–P75), and **(C)** highest (P > 75) rate of change (RoC) in the total sample, with the average trajectory being indicated by the thick black line in each of the RoC groups.

**FIGURE 3:**
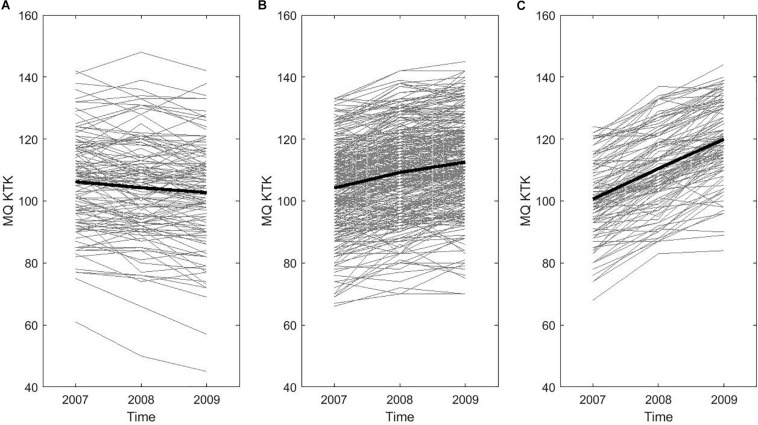
Individual trajectories in motor competence (MC) over time based on the total KTK motor quotient (MQ): Representation of **(A)** the lowest (<P25), **(B)** average (P25–P75), and **(C)** highest (P > 75) rate of change (RoC) in the total sample, with the average trajectory being indicated by the thick black line in each of the RoC groups.

Figures were also produced to illustrate individual trajectories of change in motor competence. To this end, children were divided into three groups based on their change in motor competence over time (i.e., difference in score between time point 1 and time point 3): low rate of change group (<P25), average rate of change group (P25–P75), and high rate of change group (>P75). Figure 2 shows individual changes in motor competence over time based on the KTK total raw score, whereas Figure 3 displays individual changes in motor competence development based on the total MQ of the KTK.

## Results

Table 1 shows the means and standard deviations of weight status at baseline (i.e., BMI), fitness scores at baseline (i.e., 20 m SR, SBJ, and SAR) and levels of motor competence (total raw scores on the KTK) at each time point for boys and girls separately as well as for the total sample. Motor competence generally increased over time, which is also visualized by the thick black lines in Figure 2 (change in total raw score) and Figure 3 (change in total MQ), representing the average trajectory in the low, average and high rate of change group. Both figures show the variability in individual change of motor competence for the total sample visualized by the thin lines, representing the individual trajectory of motor competence across time.

**TABLE 1:** Descriptive statistics of age, weight status at baseline, physical fitness scores at baseline and motor competence raw scores at each time point, in boys, girls and the total sample.

	**Boys (*N* = 293)**	**Girls (*N* = 265)**	**Total Sample (*N* = 558)**
**Age (years)**	8.2 ± 1.09	8.1 ± 1.15	8.2 ± 1.1
**Weight status (kg/m^2^) at baseline**	16.21 ± 2.01	16.32 ± 2.16	16.26 ± 2.08
**Physical fitness at baseline**			
Cardiorespiratory fitness: 20 m SR (min)	4.85 ± 2.22	3.55 ± 1.73	4.23 ± 2.10
Musculoskeletal fitness: SBJ (cm)	124.11 ± 20.49	118.83 ± 20.62	121.61 ± 20.70
Flexibility: SAR (cm)	19.51 ± 5.18	22.57 ± 4.93	20.96 ± 5.28
**Motor competence at each time point**			
KTKTOTAL T1 (RAW SCORE)	166.10 ± 39.84	162.89 ± 43.01	164.57 ± 41.37
KTKTOTAL T2 (RAW SCORE)	196.24 ± 40.17	191.52 ± 41.00	194.00 ± 40.60
KTKTOTAL T3 (RAW SCORE)	224.85 ± 40.70	216.99 ± 42.23	221.12 ± 41.59

*SR, shuttle run; SBJ, standing broad jump; SAR, sit and reach; KTK, Körperkoördinationstest für Kinder.*

The results of the LGCA are reported in Table 2. The LGCMs with random intercepts and slopes demonstrated good model fit (RMSEA ≤ 0.073; SRMR ≤ 0.014; CFI ≥ 0.994). Based on the total raw scores on the KTK, the analyses showed a positive linear change in motor competence over time (β = 28.48, *p* < 0.001) with significant variance in this change (*p* < 0.001). There was no significant relationship between motor competence at baseline and change in motor competence over time, based on the overall total raw scores on the KTK (*p* = 0.33).

**TABLE 2:** Results of the latent growth curve analyses.

	**Model 1**	**Model 2**	**Model 3**	**Model 4**	**Model 5**	**Model 6**	**Model 7**
**Intercept mean**	192.53^∗∗∗^	192.53^∗∗∗^	165.16^∗∗∗^	165.33^∗∗∗^	165.19^∗∗∗^	165.20^∗∗∗^	165.20^∗∗∗^
Sex				−0.36*n*.*s*.	−0.33*n*.*s*.	−0.25*n*.*s*.	−0.25*n*.*s*.
Age				1.89^∗∗∗^	1.90^∗∗∗^	1.90^∗∗∗^	1.90^∗∗∗^
Weight status					–9.97^∗∗∗^	–3.66^∗∗^	–3.67^∗∗^
Cardiorespiratory fitness						8.40^∗∗∗^	8.08^∗∗∗^
Musculoskeletal fitness						15.38^∗∗∗^	15.51^∗∗∗^
Flexibility						1.98*n*.*s*	
Intercept variance		1191.05^∗∗∗^	1583.02^∗∗∗^	943.15^∗∗∗^	859.66^∗∗∗^	543.65^∗∗∗^	546.97^∗∗∗^
Residual variance	2116.59	925.55	131.13	131.13	130.84	130.63	130.63
**Slope mean**			27.37^∗∗∗^	28.41^∗∗∗^	28.47^∗∗∗^	28.48^∗∗∗^	28.48^∗∗∗^
Sex				–2.19^∗∗^	–2.22^∗∗∗^	−2.12^∗^	−2.12^∗^
Age				–0.29^∗∗∗^	–0.0^∗∗∗^	–0.30^∗∗∗^	–0.3^∗∗∗^
Weight status					–1.38^∗∗∗^	–1.76^∗∗∗^	–1.42^∗∗^
Cardiorespiratory fitness						−0.49*n*.*s*.	
Musculoskeletal fitness						−0.72*n*.*s*.	
Flexibility						0.34*n*.*s*.	
Slope variance			45.30^∗∗∗^	29.52^∗∗∗^	28.21^∗∗∗^	26.76^∗∗∗^	27.50^∗∗∗^
Covariance			–86.23^∗∗∗^	10.17*n*.*s*.	−1.75*n*.*s*.	10.96*n*.*s*.	11.10*n*.*s*.

χ^2^	2407.75	1905.34	11.88	15.80	16.11	22.87	23.01
Df	7	6	3	5	6	9	10
RMSEA	0.784	0.753	0.073	0.062	0.055	0.053	0.049
SRMR	0.634	0.380	0.014	0.010	0.009	0.007	0.011
CFI	0.000	0.027	0.995	0.995	0.996	0.994	0.995

*^∗^*p* < 0.05; ^∗∗^*p* < 0.01; ^∗∗∗^*p* < 0.001; *n.s.*, not significant.*

Sex was not a predictor of differences in the KTK total raw score at baseline but was negatively associated with change in motor competence across 2 years; girls made less progress in motor competence than boys (β = –2.12, *p* = 0.01). Age was significantly related to the KTK total raw score at baseline, with older children demonstrating higher motor competence at baseline (β = 1.90, *p* < 0.001). Additionally, age at baseline was negatively associated with change in motor competence across time. Children who were older at baseline demonstrated less change in motor competence across 2 years (β = –0.33, *p* < 0.001). When considering the intercept and slope variance, 40.4 and 34.8% was explained by sex and age.

Children’s weight status at baseline was negatively associated with the KTK total raw score at baseline as well as with the change in motor competence across 2 years. A higher BMI level at baseline was associated with decreased motor competence (β = –3.67, *p* = 0.004). Similarly, a higher BMI at baseline was inversely related to motor competence change over time (β = –1.418, *p* = 0.002). After accounting for sex and age, weight status explained 8.9 and 4.5% of the intercept and slope variance, respectively.

Of the physical fitness outcomes, baseline levels of 20 m SR (β = 8.08, *p* < 0.001) and SBJ (β = 15.51, *p* < 0.001) were directly related to motor competence levels at baseline. After accounting for sex, age, and BMI, both 20 m SR and SBJ explained 36.8% of the intercept variance. When considering change in motor competence over time, none of the physical fitness components measured at baseline was shown to be significantly associated.

## Discussion

The purpose of this longitudinal study was to gain more insight into developmental change in children’s motor competence over time and to investigate the potential influence of weight status and physical fitness on that change. Therefore, a LGCA was conducted to investigate the developmental change in motor competence. This approach is appropriate as it models each child’s trajectory of change in motor competence across time. It considers differences in developmental trajectories across children and the potential influence of baseline weight status and physical fitness on this individual change.

Consistent with previous research (e.g., Ahnert et al., 2009; Vandorpe et al., 2011; Dos Santos et al., 2018), an average positive change in motor competence over 2 years was found. Yet, the results of the LGCA revealed that there was significant variance in how children’s motor competence develops over time, which is also consistent with Dos Santos et al. (2018), and clearly illustrated in both Figures 2, 3 of the present paper.

Visual inspection of Figure 2 shows a large variability in the rate of change across the current sample. In some children, the overall motor competence raw score demonstrates improvement in a linear fashion over the 2 years, whereas others show little change after 1 year followed by an improvement in year 2, and still others show an increase in year 1 that levels off in year 2. It is interesting to note that when these raw scores are converted to age- and sex-adjusted MQs as displayed in Figure 3, a number of children actually stagnated (1.1%) or demonstrated a delayed development (14.7%) of motor competence compared to the reference sample (Figure 3; Kiphard and Schilling, 2017). MQ is considered a relatively stable construct over time for the average child (Vandorpe et al., 2011), but results of the LGCA demonstrate there was statistically significant variability in trajectories of change in motor competence among individual children. Whilst an improvement in raw scores on the KTK was present in virtually every child in the sample (99.6%; see Figure 2), this improvement may be considered insufficient to keep up with the expected motor development, which is evident from Figure 3, where only 39.0% makes progress, with respect to age- and sex-related norms.

Regarding the level of motor competence at baseline, the LGCA showed that there was no significant relationship between motor competence at baseline and the change in motor competence over time. Indeed, inspection of Figures 2 and 3 shows that each rate of change group included children with a high(er) or low(er) level of motor competence at baseline. Our results thus suggest that each child can improve his/her level of motor competence over a period of 2 years, regardless of his/her initial level of motor competence. The finding of inter-individual variation in motor competence development is in agreement with the study of Rodrigues et al. (2016), where children also demonstrated divergent developmental pathways in fitness across childhood. Interestingly, the study by Rodrigues et al. (2016) reported that many children in the low rate of change group did not change in raw performance or actually decreased in raw performance over time, whereas the present study demonstrated a general positive change in motor competence over time irrespective of the level of motor competence at baseline. In contrast, Dos Santos et al. (2018) found that higher levels of motor competence at 6 years of age demonstrated lower rate of change over time. It should be noted that both studies included samples of one age group or grade (i.e., 6 years/grade 1) followed over time, whilst the present study sample covered a larger age range at baseline (i.e., 6–9 years). Additionally, Rodrigues et al. (2016) mainly focused on components of fitness rather than motor competence (i.e., SLJ, 50 m dash, 10 m SR, 60 s sit-ups, flexed arm hang, SAR, 20 m SR). The extent to which children can improve their motor competence level and redirect their trajectories later on, remains a pertinent question and should be further explored.

The present findings showed that there was no significant difference in motor competence between boys and girls at baseline. Most previous studies have reported sex differences in favor of boys in this age range although different results have been found across specific motor domains. In their systematic review, Barnett et al. (2016) found strong evidence for boys scoring better on motor coordination compared to girls whilst reporting inconclusive evidence for girls outperforming boys on stability measures. As the KTK covers both aspects of motor coordination and dynamic balance, this might explain the divergent finding in the present study. Interestingly, boys in our sample made more progress in motor competence over time compared to girls. Prior research has generally not specifically investigated how sex influences motor competence development. The study of Dos Santos et al. (2018), however, found differences in the trajectory of change favoring girls whilst the study of Rodrigues et al. (2016) found no differences. In light of these contrasting findings, there is clearly a need for more research into how boys and girls develop their motor competence levels over time and how this might be (differently) affected by factors such as physical activity participation and sports preferences.

In alignment with previous literature (e.g., Barnett et al., 2016), the present study results showed a positive relationship between age and the level of motor competence at baseline. However, our data indicate that as age increases, change in motor competence decreases. Although data on this topic in literature is limited, it is generally assumed that early childhood is marked by major changes in physical and motor development (Gallahue et al., 2012). However, as noted by Gallahue and Ozmunn (2005), middle childhood is characterized by “slow but steady increases in height and weight and progress toward greater organization of the sensory and motor systems” (p. 178). Although older children still make progress in their motor competence, these findings do seem to support early interventions focused on developing motor competence at a younger age. This, in turn, will help children to successfully participate in sports, games and other types of physical activity as they grow older.

Another purpose of this study was to examine if weight status and physical fitness influenced children’s individual trajectory of change in motor competence over time. Results revealed a significant inverse relationship between weight status and motor competence at baseline, which is in agreement with previous research (Martins et al., 2010; Lopes et al., 2012; D’Hondt et al., 2013, 2014; Cattuzzo et al., 2016; Rodrigues et al., 2016; Lima et al., 2018). Moreover, children’s baseline weight status was inversely associated with change in motor competence. Specifically, a higher weight status at baseline was associated with less progress in motor competence. This partly supports Stodden et al. (2008) notion of a negative spiral of disengagement where children with a less optimal weight status are at greater risk to end up in becoming less motor competent over time, which may lead to reduced physical activity participation and lower physical fitness.

With respect to physical fitness, it was indeed shown that baseline levels of cardiorespiratory and musculoskeletal fitness were significantly related to motor competence at baseline, which is consistent with findings from earlier studies (Lubans et al., 2010; Cattuzzo et al., 2016; Utesch et al., 2019). However, no significant relationship between trunk flexibility and motor competence at baseline was found. Contrary to these findings, Lopes et al. (2017) found a positive association between flexibility and motor competence in children. It should be noted that there is an age difference between the sample of the present study (6–9 years) and that of the study of Lopes et al. (2017; 9–12 years). More research is warranted to further understand the association between motor competence and flexibility as there is currently limited evidence available on this relationship (Cattuzzo et al., 2016; Utesch et al., 2019). Although physical fitness is considered an important marker of current and future health in both children and adults, none of the components of physical fitness (i.e., cardiorespiratory fitness, musculoskeletal fitness and flexibility) were significantly associated with change in motor competence over time. This is in contrast with the study by Dos Santos et al. (2018), who found that children in the age range of 6–9 years with higher levels of physical fitness demonstrated higher scores on motor competence across a 4-year timespan. It should be noted that our study particularly focused on how physical fitness at baseline influenced change in motor competence over time. In light of the limited longitudinal evidence on this topic, there is a need for more research investigating the role that different physical fitness components may have on motor competence development across childhood.

The present study investigated children’s trajectories of change in motor competence across 2 years and explored the influence of baseline weight status and physical fitness on these trajectories. The longitudinal design and the use of LGCA are major strengths of the current study. Using this statistical approach allows for the estimation of inter-individual variability in intra-individual trajectories of change over time, whereas more traditional methods for analyzing repeated measures data are more limited in this respect. However, some limitations need to be addressed. First, the present study only investigated linear change in motor competence as data were only collected across three time points. However, considering the variability in individual trajectories (see Figure 2) across a longer time frame should be investigated and may demonstrate non-linear change in motor competence across time (i.e., including ≥4 time points). This type of analysis will provide a more comprehensive understanding of childhood developmental pathways of motor competence. Second, children’s BMI was used as the sole indicator of weight status. As BMI is an indirect estimate of adiposity, further investigations should include additional anthropometric measures, such as waist circumference and skinfolds or more advanced techniques (such as Bioelectrical Impedance Analysis (BIA) or Dual-energy X-ray Absorptiometry (DXA) to better estimate weight status and/or fat percentage. Third, the present study focused solely on gross motor coordination and did not examine other behavioral attributes such as physical activity and sport participation. For this reason, it is impossible to determine if the variability in intra-individual trajectories of change over time is related to sports practice or physical activity participation. As motor competence is associated with many health-related outcomes, more research is recommended to explore how other components of motor competence change over time and how this variation is linked with both physiological and psychological factors (e.g., physical fitness, weight status, perceived competence, and motivation) as well as behavioral and environmental factors (e.g., physical activity, and socioeconomic background). As noted by Robinson et al. (2015), children’s development is “a complex and multifaceted process that synergistically evolves across time” (p. 1273). Barnett et al. (2016) determined that child-level variables such as age, sex, weight status, physical activity, fitness, and socioeconomic background are all important individual correlates of motor competence. Therefore, future longitudinal studies are recommended to further explore the potential role of such (additional) correlates in order to gain more insight in the mechanisms underlying children’s individual trajectories of motor competence across time.

## Conclusion

In summary, this 2-year longitudinal follow-up study demonstrates a general positive linear change in children’s motor competence over time, although there is significant variance in trajectories among individuals. Moreover, the level of motor competence at baseline was not found to be associated with change in motor competence over time. Our findings call for a shift toward a person-centered developmental approach for understanding change in motor competence development. This study further showed that weight status is not only negatively associated with motor competence at baseline, but it also negatively influences change in motor competence across childhood. This suggests that overweight children are at higher risk of making less positive change in motor competence over time. Additionally, whilst both cardiorespiratory and musculoskeletal fitness were positively related to motor competence at baseline, they did not significantly affect change in motor competence over time. Our findings highlight the importance of exploring individual change in motor competence across childhood in order to develop more effective movement programs and to better support children’s motor development.

## Data Availability Statement

The raw data supporting the conclusions of this manuscript will be made available by the authors, without undue reservation, to any qualified researcher.

## Ethics Statement

This study was approved and carried out in accordance with the recommendations of the Ethics Committee of Ghent University Hospital. All participants and their parents/legal guardians gave written informed consent in accordance with the Declaration of Helsinki.

## Author Contributions

EC, FB, DS, and ML were involved in the conceptualization of the study. EC, FB, and ML analyzed data of the manuscript. All authors wrote the manuscript and approved the final version of the manuscript.

## Conflict of Interest

The authors declare that the research was conducted in the absence of any commercial or financial relationships that could be construed as a potential conflict of interest.
